# CRISP-view: a database of functional genetic screens spanning multiple phenotypes

**DOI:** 10.1093/nar/gkaa809

**Published:** 2020-10-03

**Authors:** Yingbo Cui, Xiaolong Cheng, Qing Chen, Bicna Song, Anthony Chiu, Yuan Gao, Tyson Dawson, Lumen Chao, Wubing Zhang, Dian Li, Zexiang Zeng, Jijun Yu, Zexu Li, Teng Fei, Shaoliang Peng, Wei Li

**Affiliations:** Sanyi Road, Changsha, Hunan Province, People's Republic of China; Center for Genetic Medicine Research, Children's National Hospital. 111 Michigan Ave NW, Washington, DC 20010, USA; Center for Genetic Medicine Research, Children's National Hospital. 111 Michigan Ave NW, Washington, DC 20010, USA; Center for Genetic Medicine Research, Children's National Hospital. 111 Michigan Ave NW, Washington, DC 20010, USA; Center for Genetic Medicine Research, Children's National Hospital. 111 Michigan Ave NW, Washington, DC 20010, USA; School of Medicine and Health Sciences, George Washington University, 2300 I Street NW, Washington, DC 20037, USA; Center for Genetic Medicine Research, Children's National Hospital. 111 Michigan Ave NW, Washington, DC 20010, USA; Department of Biochemistry and Molecular Biology, George Washington University, 2300 I Street NW, Washington, DC 20037, USA; Center for Genetic Medicine Research, Children's National Hospital. 111 Michigan Ave NW, Washington, DC 20010, USA; Institute for Biomedical Sciences, George Washington University, 2300 I Street NW, Washington, DC 20037, USA; Computational Biology Institute, Milken Institute School of Public Health, George Washington University, 45085 University Drive, Ashburn, VA 20148, USA; Center for Genetic Medicine Research, Children's National Hospital. 111 Michigan Ave NW, Washington, DC 20010, USA; Department of Data Sciences, Dana-Farber Cancer Institute and Harvard T.H. Chan School of Public Health. 450 Brookline Ave., Boston MA 02215, USA; Department of Data Sciences, Dana-Farber Cancer Institute and Harvard T.H. Chan School of Public Health. 450 Brookline Ave., Boston MA 02215, USA; Department of Data Sciences, Dana-Farber Cancer Institute and Harvard T.H. Chan School of Public Health. 450 Brookline Ave., Boston MA 02215, USA; Beijing Key Laboratory of Therapeutic Gene Engineering Antibody. Beijing, People's Republic of China; College of Life and Health Sciences, Northeastern University. 110819 Shenyang, People's Republic of China; College of Life and Health Sciences, Northeastern University. 110819 Shenyang, People's Republic of China; Lushan South Road, Changsha, Hunan Province, People's Republic of China; Center for Genetic Medicine Research, Children's National Hospital. 111 Michigan Ave NW, Washington, DC 20010, USA; Department of Genomics and Precision Medicine, George Washington University. 111 Michigan Ave NW, Washington, DC 20010, USA

## Abstract

High-throughput genetic screening based on CRISPR/Cas9 or RNA-interference (RNAi) enables the exploration of genes associated with the phenotype of interest on a large scale. The rapid accumulation of public available genetic screening data provides a wealth of knowledge about genotype-to-phenotype relationships and a valuable resource for the systematic analysis of gene functions. Here we present CRISP-view, a comprehensive database of CRISPR/Cas9 and RNAi screening datasets that span multiple phenotypes, including *in vitro* and *in vivo* cell proliferation and viability, response to cancer immunotherapy, virus response, protein expression, etc. By 22 September 2020, CRISP-view has collected 10 321 human samples and 825 mouse samples from 167 papers. All the datasets have been curated, annotated, and processed by a standard MAGeCK-VISPR analysis pipeline with quality control (QC) metrics. We also developed a user-friendly webserver to visualize, explore, and search these datasets. The webserver is freely available at http://crispview.weililab.org.

## INTRODUCTION

Functional genetic screening is a high-throughput, cost-effective technology to identify genes or genomic elements that are pertinent to a phenotype-of-interest (1–7). Based on RNA-interference (RNAi) or CRISPR/Cas9 (8–11), screening explores the functions of genes (or non-coding elements) in various contexts including cancer progression, interaction with immune system, response to drug treatment, virus infection etc. (12–17). The broad spectrum of possible phenotypes that can be covered by genetic screening provides a wealth of information on our understanding of the genes, non-coding elements, and their associated pathways in different aspects (18–25).

With the rapid accumulation of genetic screens in recent years, several databases are developed to collect, visualize and compare these datasets. GenomeCRISPR (26) collects human cell-line CRISPR/Cas9 screening datasets and enables users to explore the behavior of genes and sgRNAs. PICKLES (27) enables pooled *in vitro* knock-out CRISPR screening data to be visualized together with other types of data including copy number variation, expression and mutation. BioGRID ORCS (28) reports scores of CRISPR/Cas9 screening, generated by different analysis algorithms, from the original publication. In addition, large-scale genome-wide screening projects, including Project Drive (29), DepMap (30) and Sanger DepMap (or Project Score) (31), provided a centralized web interface for users to browse *in vitro* screening data as well as the associated genomic profiles from hundreds of cell lines. However, several limitations restrict the wide application of these datasets. For example, all these databases collected screens that mainly measure the *in vitro* proliferation (or viability) of cancer cell lines, while screens of other phenotypes are lacking. Among those, DepMap and Sanger DepMap only included in-house screening datasets on cancer cell lines. Other datasets only stored published analysis results of screens reported in the paper (like BioGRID ORCS, GenomeCRISPR), or do not include up-to-date screening datasets. As the analysis results are generated by different methods from different studies, and the quality of the datasets may vary, it is difficult to systematically compare screens across different datasets. Therefore, a central challenge is to store, process, and evaluate genetic screening data spanning multiple phenotypes and in a standardized way, where users are able to gain new biological insights through data mining.

Here we present CRISP-view, a database of CRISPR- or RNAi-based genetic screening spanning various phenotypes, including *in vitro* and *in vivo* cell proliferation or viability, immune or immunotherapy response, virus infection, protein expression (by GFP sorting), etc. As of 22 September 2020, CRISP-view collected 11,146 samples with unified metadata annotation, curation and standardized quality control metrics. CRISP-view also provides a web interface for users to search and browse all the datasets (and their associated metadata). CRISP-view represents the most comprehensive collection of screening datasets up to date, and is constantly being updated as screening datasets accumulates in the public domain.

## DATABASE CONTENT

**Figure 1.**
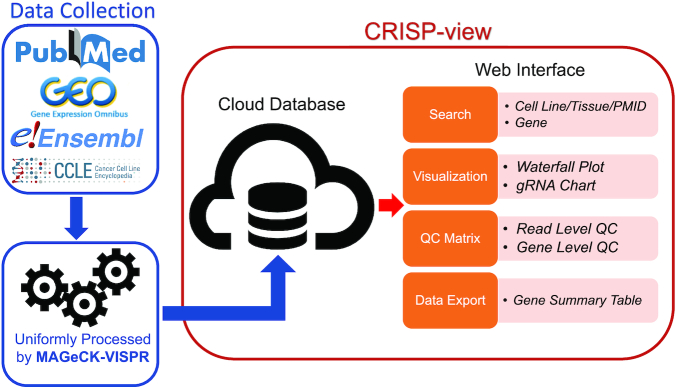
A Schematic view of CRISP-view. The CRISP-view database consists of three parts: a curated metadata collection, a unified screening data processing and a web interface.

CRISP-view is a comprehensive annotated resource of public genetic screening data in human and mouse. CRISP-view contains >11 000 genetic *in vitro* and *in vivo* screening samples from 167 (and growing) publications. A variety of different screening technologies are covered, including CRISPR activation (CRISPRa), CRISPR inhibition (CRISPRi), CRISPR knockout and RNA interference (RNAi). The CRISP-view database consists of three parts: a curated metadata collection, unified screening data processing and a web interface. All data sources and web interface features are summarized in Figure 1.

## DATA SOURCES AND METADATA ANNOTATION

CRISP-view collects publicly available CRISPR/Cas9 and RNAi screening data from gene expression omnibus (GEO), the supplemental materials of papers or from the author directly. We systematically annotated the metadata of these samples manually, including species, screening type, cell line, library, screening conditions, associated PubMed ID and citation.

**Figure 2.**
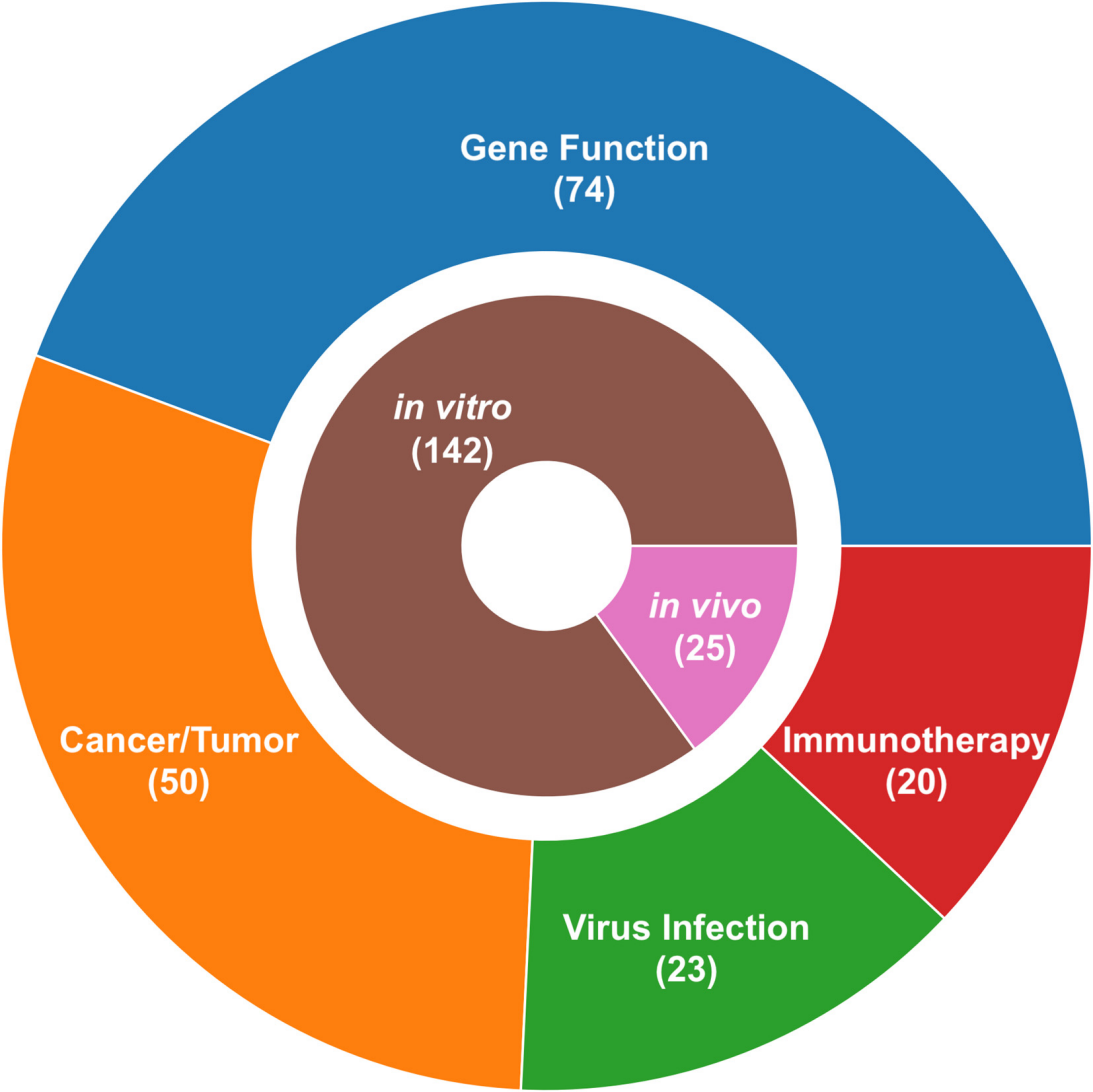
Statistics of datasets collected in CRISP-view so far. The dataset includes *in vitro* and *in vivo* experiments and covers a variety of research areas, including gene function, cancer/tumor, Immunotherapy and virus infection.

In total, our database contains 11 146 samples from 167 high-throughput genetic experiments, including 10 321 (and 825) human (and mouse) samples, respectively. Included in the database are 1271 human and 29 mouse cell line screens, respectively. There are 81 different screening libraries used in these high-throughput experiments. The database also covers a very wide range of research subjects, including proliferation and viability in cancer cells and mouse models, immune and immunotherapy related screens, virus related screens, and the expression of certain gene marker (Figure 2 and Supplementary Figure S1). We list all the publications incorporated in CRISP-view database in the Statistics Page (http://crispview.weililab.org/statistics).

## DATA PROCESSING AND QUALITY CONTROL METRICS

To ensure the consistency between different datasets, we limit our screening datasets to those where raw sequence data (fastq format) or raw count tables are available. The data is uniformly processed by the MAGeCK-VISPR pipeline (32,33) that generates QC measurements and beta scores for all the perturbed genes. Beta-score is a measurement to reflect the functions of genes in the screen, similar to the term ‘log fold change’ in differential expression analysis: a positive (or negative) beta score indicates a positive (or negative) selection of the corresponding gene in the screen, respectively.

The QC measurement, generated by MAGeCK-VISPR, includes the number of reads and the percentage of mapped reads, the number of sgRNAs with zero read count, and the Gini index of read count distribution. In addition, we evaluate the degree of negative selection on ribosomal genes using GSEA (34) as a measurement of quality in proliferation-based dropout screens, because the knockout of ribosomal genes is expected to have a strong negative selection phenotype (1,35). We also define a threshold for each QC metric for pass or fail, based on the distribution of that metric in all samples. A QC metric is considered as pass if it is better than 2/3 of the samples in the database.

## DATABASE INTERFACE AND TUTORIAL

The CRISP-view website is available at http://crispview.weililab.org. The main page provides options for users to select species and screening technologies, and to search datasets by gene symbol, publication ID (PMID), treatment conditions or biological source (cell line name or tissue type).

**Figure 3.**
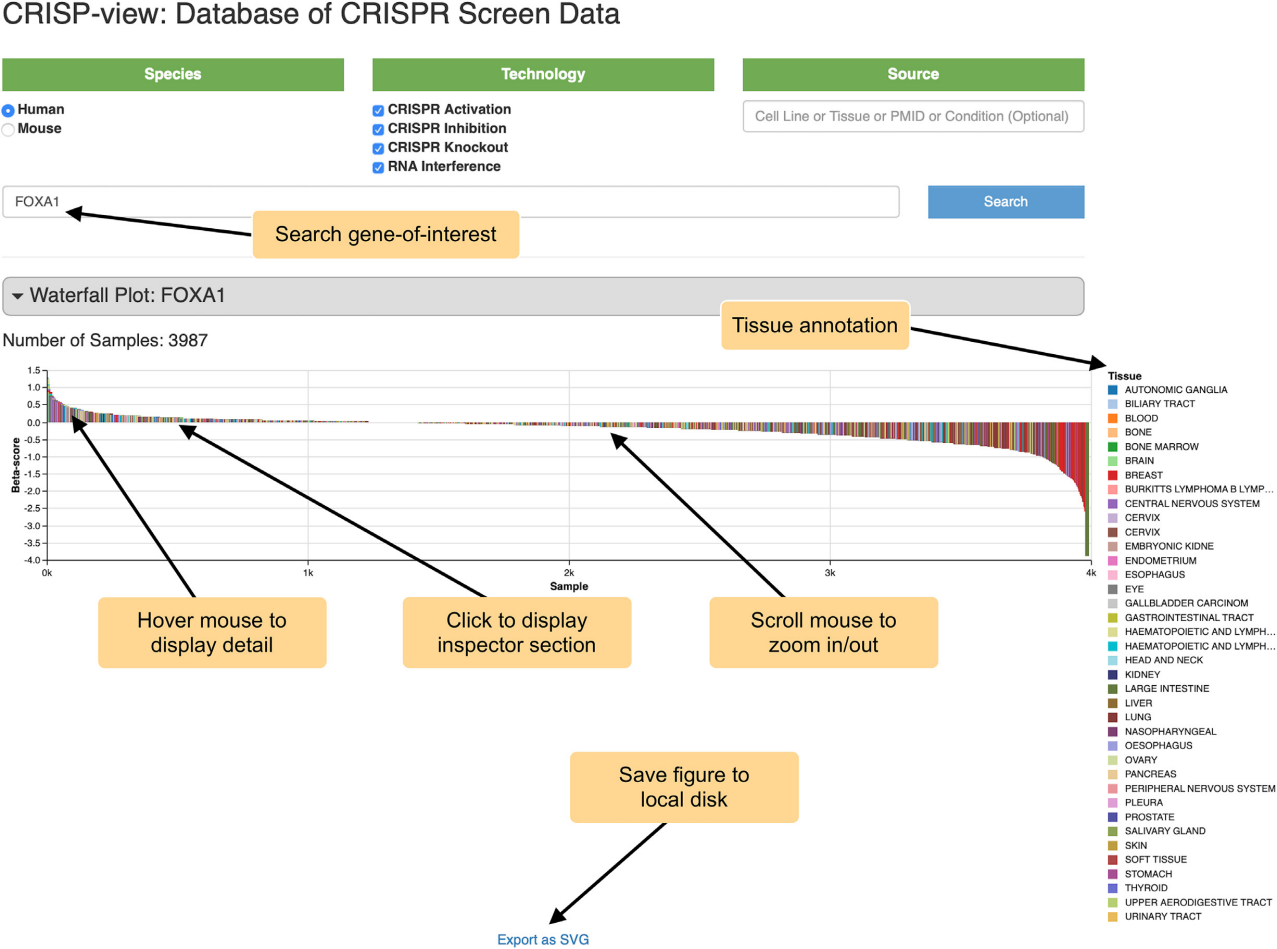
Searching genes of interest in CRISP-view. FOXA1 is shown as an example and a waterfall plot is shown below. Different colours in the waterfall plot represent different tissues of origin of cell line.

User can explore the beta scores of interesting gene by searching gene symbols, and view detailed data annotations, quality control metric and positively/negatively selected genes of individual sample. After entering a gene symbol, a waterfall plot will display the ranked beta-score of that gene in all samples (Figure 3). Different colors in waterfall plot represent different tissues of origin for cell lines. The waterfall plot allows users to zoom-in, zoom-out or move within the image for exploration. When mouse hovers over the waterfall plot, a smaller view window will show the basic information of the selected sample (e.g. cell line name, tissue of origin, screening library, treatment condition, the beta-score, and source). After clicking on any data point in waterfall plot, users can navigate the inspector section (behind the waterfall plot) to inspect the corresponding sample in more details. The waterfall plot can be saved locally in SVG format.

**Figure 4.**
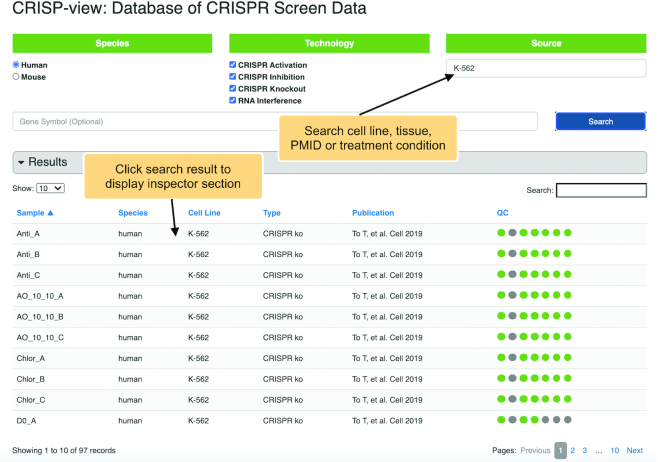
Searching in CRISP-view. ‘K-562’ is used as an example of search term and CRISP-view returns all matched samples. The corresponding sample information and QC metrics are listed in the table.

**Figure 5.**
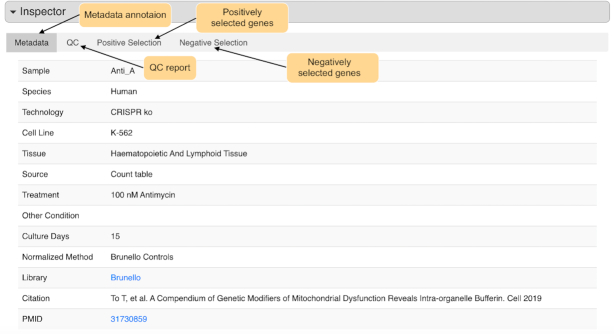
Metadata Inspector view of individual sample. The inspector section displays the detailed data annotations, quality control report and positively/negatively selected genes of individual sample in separated tabs.

**Figure 6.**
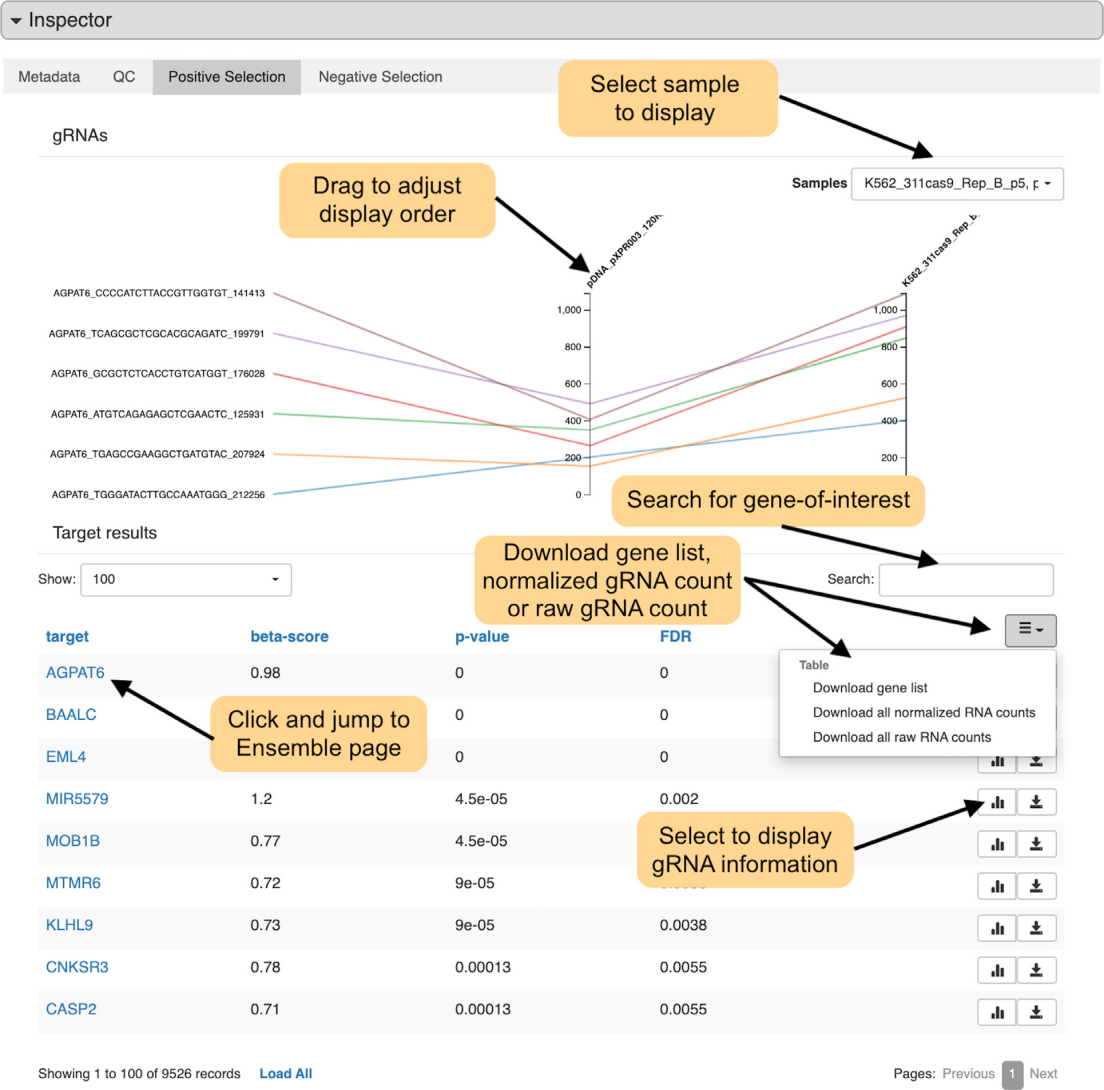
Positive selection Inspector view of individual sample.

User can also search interesting cell line, tissue, publication or treatment condition, where matched samples will be shown in a table (Figure 4). User can view the detailed information of each sample in the inspector section (Figure 5). In the inspector section, CRISP-view provides three layers of content for each sample in four tabs: a manually curated metadata annotation (first tab), QC results (second tab) and a list of positively and negatively selected genes and associated sgRNAs (third and fourth tabs). Metadata annotation includes sample name, species, screening technology, cell line, tissue of origin, source data format, normalized method, screening library and citation. In the QC results, the quality control report is shown briefly by colored circles: green (and red) indicates the metric passed (or failed) the threshold, respectively. A list of positively (and negatively) selected genes and associated sgRNAs are shown in the third (and fourth) tabs, respectively, where genes are ranked according to their beta-scores (Figure 6). User can view normalized read counts of gRNAs in selected sample and its corresponding initial condition(s), and download gene selection information as tab-separated text file.

## IMPLEMENTATION

The client of the database is implemented in jQuery and Twitter Bootstraps, and the server is implemented with Apache in AWS EC2. The communication between servers and clients uses JSON format (by Ajax), which efficiently reduces response time and improves user experience. In order to build a robust and scalable database, all the datasets are stored in MySQL database with AWS Rational Database Service (RDS) that contains thousands of samples and millions of data points (gene beta scores and sgRNA normalized counts). We used several strategies to speed up the database. First, we use the Ajax web model that allows the server to send or receive data asynchronously in the background, without interfering with the display of the client. Second, the client only loads the top 100 positively or negatively selected genes by default, and will load additional genes per user's request. Third, we constructed a multi-column index for the MySQL database to enable quick search over values of different columns. This strategy efficiently reduces the amount of data transferring to the client by default and improves the speed of inquiry.

## DISCUSSION AND FURTHER DIRECTIONS

We present CRISP-view, a comprehensive annotated database of RNAi and CRISPR/Cas9 genetic screens in human and mouse samples. All the collected raw data is processed by a uniform MAGeCK-VISPR pipeline with complete metadata annotation and QC measurements. CRISP-view provides a web interface to visualize the datasets and associated metadata, and enables researchers to explore interesting genes, cell lines, tissues, studies or conditions. The CRISP-view database is updated on a regular basis to incorporate newly published genetic screening data, providing a powerful resource for researchers to explore gene functions associated with different phenotypes.

Future works of CRISP-view include data mining and machine learning approaches to better understand the gene functions in different datasets or conditions, and more visualization and analysis tools to associate screening data with other types of data (like expression, mutation, copy number variation, and epigenetic profiles).

## Supplementary Material

gkaa809_Supplemental_File
