# Supplementary material for: CRISP-view: a database of functional genetic screens spanning multiple phenotypes
Source: Nucleic Acids Res. 2020 Oct 3;49(D1):D848–54. doi: 10.1093/nar/gkaa809 (PMC7778972; doi:10.1093/nar/gkaa809)
Supplement: gkaa809_Supplemental_File [file gkaa809_supplemental_file.pdf]

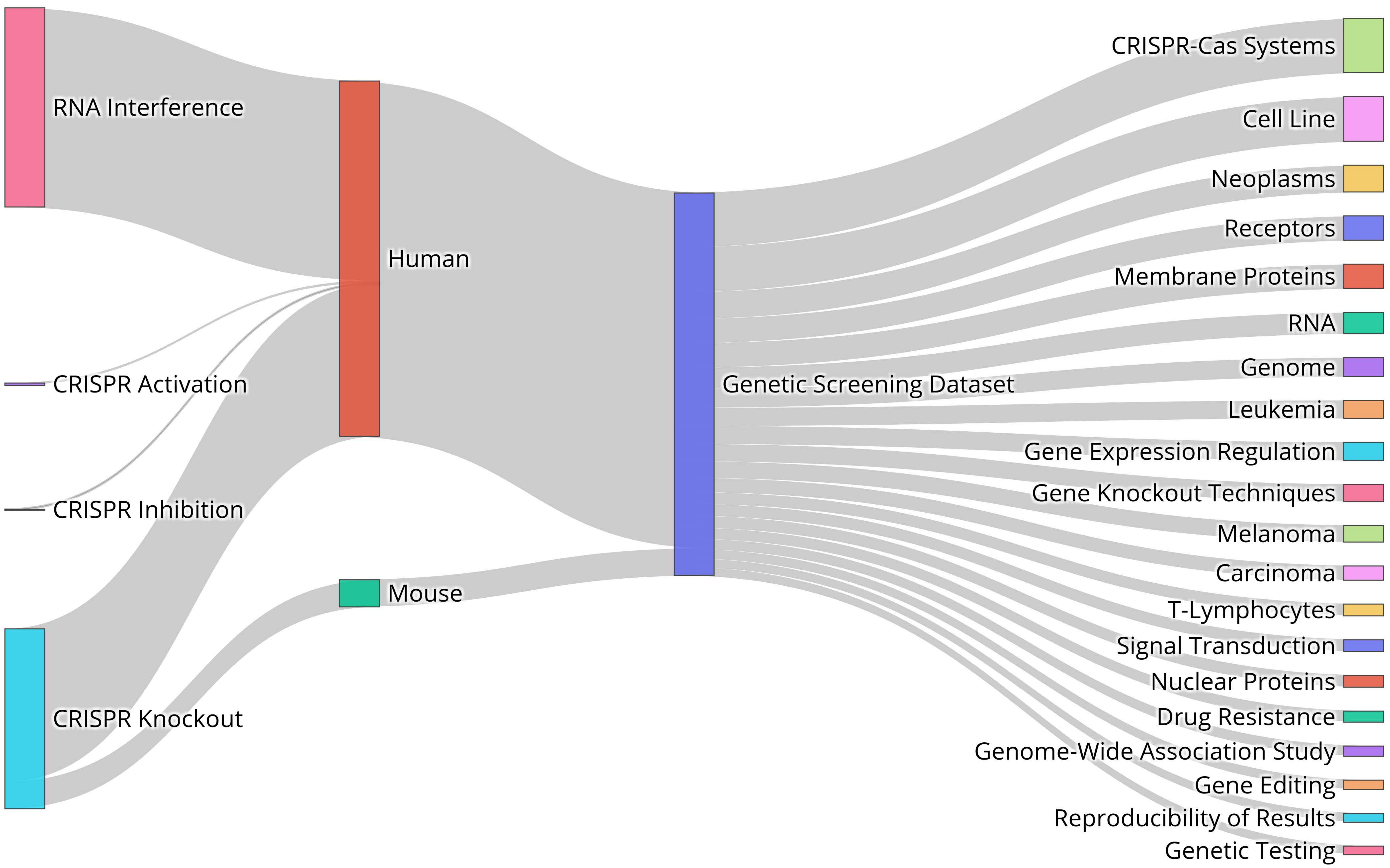

Figure S1. Database sample statistics. The two leftmost columns indicate the sample classification, while the rightmost column show the subject headings of the genetic screening datasets based on NCBI MeSH.
